# *In Vivo* T Cell Activation Induces the Formation of CD209^+^ PDL-2^+^ Dendritic Cells

**DOI:** 10.1371/journal.pone.0076258

**Published:** 2013-10-02

**Authors:** Matthew G. Davidson, Michael N. Alonso, Justin A. Kenkel, Megan M. Suhoski, Joseph C. González, Robert Yuan, Edgar G. Engleman

**Affiliations:** Department of Pathology, Stanford University School of Medicine (Blood Center), Palo Alto, California, United States of America; New York University, United States of America

## Abstract

Two critical functions of dendritic cells (DC) are to activate and functionally polarize T cells. Activated T cells can, in turn, influence DC maturation, although their effect on *de novo* DC development is poorly understood. Here we report that activation of T cells in mice, with either an anti-CD3 antibody or super antigen, drives the rapid formation of CD209^+^CD11b^+^CD11c^+^ MHC II^+^ DC from monocytic precursors (Mo-DC). GM-CSF is produced by T cells following activation, but surprisingly, it is not required for the formation of CD209^+^ Mo-DC. CD40L, however, is critical for the full induction of Mo-DC following T cell activation. T cell induced CD209^+^ Mo-DC are comparable to conventional CD209^-^ DC in their ability to stimulate T cell proliferation. However, in contrast to conventional CD209^-^ DC, CD209^+^ Mo-DC fail to effectively polarize T cells, as indicated by a paucity of T cell cytokine production. The inability of CD209^+^ Mo-DC to polarize T cells is partly explained by increased expression of PDL-2, since blockade of this molecule restores some polarizing capacity to the Mo-DC. These findings expand the range of signals capable of driving Mo-DC differentiation *in vivo* beyond exogenous microbial factors to include endogenous factors produced following T cell activation.

## Introduction

Monocytes serve as precursors to a variety of cell types, including macrophages, osteoclasts and inflammatory dendritic cells (DC). Their ultimate fate is dependent on the interpretation of a diverse set of environmental cues often in the form of pathogen associated molecular patterns (PAMPs). The correct interpretation of these cues by monocytes is essential for both homeostasis and the generation of productive immune responses. For example, the differentiation of monocytes into TNFα/iNOS producing DC (TipDC) is critical for clearance of *Listeria monocytogenes* infection [1].

While certain pathogens are capable of driving monocyte differentiation into DC (Mo-DC), the formation of inflammatory DC, putatively of monocytic origin, during sterile autoimmune diseases suggests that non-microbial signals also drive Mo-DC formation [2-5]. Multiple endogenous stimuli, including GM-CSF stimulation [6], migration of monocytes across an endothelial barrier [7] and CD40 ligation [8], promote Mo-DC formation *in vitro*. However, the signals that elicit Mo-DC formation *in vivo* during sterile inflammation have not been elucidated.

Since communication between DC and T cells is bidirectional and newly activated T cells are capable of maturing bystander DC [9], we considered the possibility that activated T cells might drive *de novo* DC formation from monocytes. Our group previously demonstrated that both human [10] and murine T-helper cells [11] induce Mo-DC differentiation during *in vitro* coculture experiments, but such activity has not yet been demonstrated *in vivo*. Due to the lack of a reliable marker capable of distinguishing between Mo-DC and conventional DC, it has been difficult until recently to study the *in vivo* biology of Mo-DC. Because of this, most investigators have studied the basic biology of Mo-DC by utilizing *in vitro* generated cells. Here, we took advantage of the discovery that Mo-DC, but not conventional DC (cDC), express the C-type lectin CD209 [12], to elucidate the capacity of T cells to elicit DC differentiation from monocytes *in vivo*.

## Materials and Methods

### Ethics Statement

Animal experiments were approved and conducted in accordance with Stanford University APLAC protocol #13605. All efforts were taken to minimize animal suffering.

### Mice

Female C57BL/6, GM-CSFR KO and CD40L KO mice were purchased from Jackson Labs. OT-II TCR transgenic Rag2^-/-^ mice were purchased from Taconic. All mice were housed in an American Association for the Accreditation of Laboratory Animal Care–accredited animal facility and maintained in specific pathogen-free conditions.

### In Vivo Experiments

Mice were injected intravenously with 10µg dialyzed PE conjugated anti-CD209 antibody clone 5H10 or dialyzed PE conjugated isotype control antibody (eBioscience). Immediately afterwards, mice were injected in the footpad with various doses of azide and endotoxin free anti-CD3 antibody clone 2C11 or the appropriate isotype control (eBioscience) or TSST-1 (Sigma). 16-18 hours later the draining popliteal, inguinal and axillary LNs were harvested and digested for 30 minutes at 37°C with 400U/ml collagenase IV (Worthington) in RPMI media (Gibco) supplemented with 100 U/mL penicillin, 100µg/mL streptomycin, 2mM L-glutamine and 10% fetal calf serum.

### Magnetic Cell Separation (MACS)

T cells were purified from LN suspensions with biotinylated anti-CD4 clone GK1.5 (Becton Dickinson) or biotinylated anti-CD8a clone 53-6.7 (Biolegend) followed by anti-biotin microbeads (Miltenyi).

### Flow Cytometry and Sorting

Single cell suspensions were FC receptor blocked and then stained with antibodies conjugated to various fluorochromes or biotin. Antibodies used included I-A/I-E (MHCII), CD69, CD209, F4/80, CD206, CD11b, CD11c, CD40, DEC-205, Thy1.2, CD19, DX-5, Ly6c, CD80, CD86, CD40, PDL-1, PDL-2, ICOSL and B7-S1. Dead cells were gated out as DAPI^+^ (Invitrogen). Cells were analyzed on a LSR-II (BD) or sorted on a FACS Aria II (BD).

### ELISA

GM-CSF in serum obtained via cardiac puncture, and IFNγ, IL-17A, IL-10 and IL-4 in cell culture supernatants were measured by ELISA (eBioscience).

### Microscopy

LN were harvested and fixed in 4% PFA + 10% sucrose in PBS for 30 minutes on ice before being embedded in OCT medium and snap frozen. 10 µm sections were air dried and then fixed in acetone at -20°C for 10 minutes. They then were stained with BMD10 hybridoma supernatant [13] followed by goat α-rat IgG HRP followed by Alexa Fluor 488 Tyramide amplification (Invitrogen) followed by a 1 hour incubation with APC conjugated B220 (Biolegend). Cells were mounted with Fluoro-Gel II with DAPI (Electron Microscopy Sciences) and imaged using a Leica 2500 confocal microscope. Sorted cells were transferred to coverslip bottom chamber slides and imaged using differential interference contrast (DIC) microscopy on a Leica DM IRB with Metamorph software (Molecular Devices).

### Antigen Specific T Cell Proliferation Assay

Sorted DC from anti-CD3 injected mice as well as B cells (CD19^+^ cells) or monocytes (CD11b^+^, Ly6c^+^) from wild type mice were sorted and pulsed with 2.5µg/ml ISQ peptide in media for 1 hour at 37°C. They were washed twice and then cocultured at various ratios with MACS purified OT-II CD4^+^ T cells from OT-II Rag KO mice in 200 µl in round bottom wells for 3 days. Thereafter the cells were pulsed with 1 μCi/well of H^3^-thymidine and cultured for an additional 18 hours before being harvested by Harvester 400 (Tomtec). Radioactivity was measured by a MicroBeta 1450 counter (LKB Wallac).

### Statistical Analyses

An unpaired student’s *t*-test (2-tailed) with a 95% confidence interval was performed in Prism (GraphPad) to analyze all experimental data unless otherwise stated. Error bars represent ^+/-^ SEM. P<0.05=*; P<0.01=**; P<0.001=***.

## Results

### T cell activation mobilizes monocytes into lymph nodes (LNs) and drives their differentiation into CD209^+^ DC

**Figure 1:**
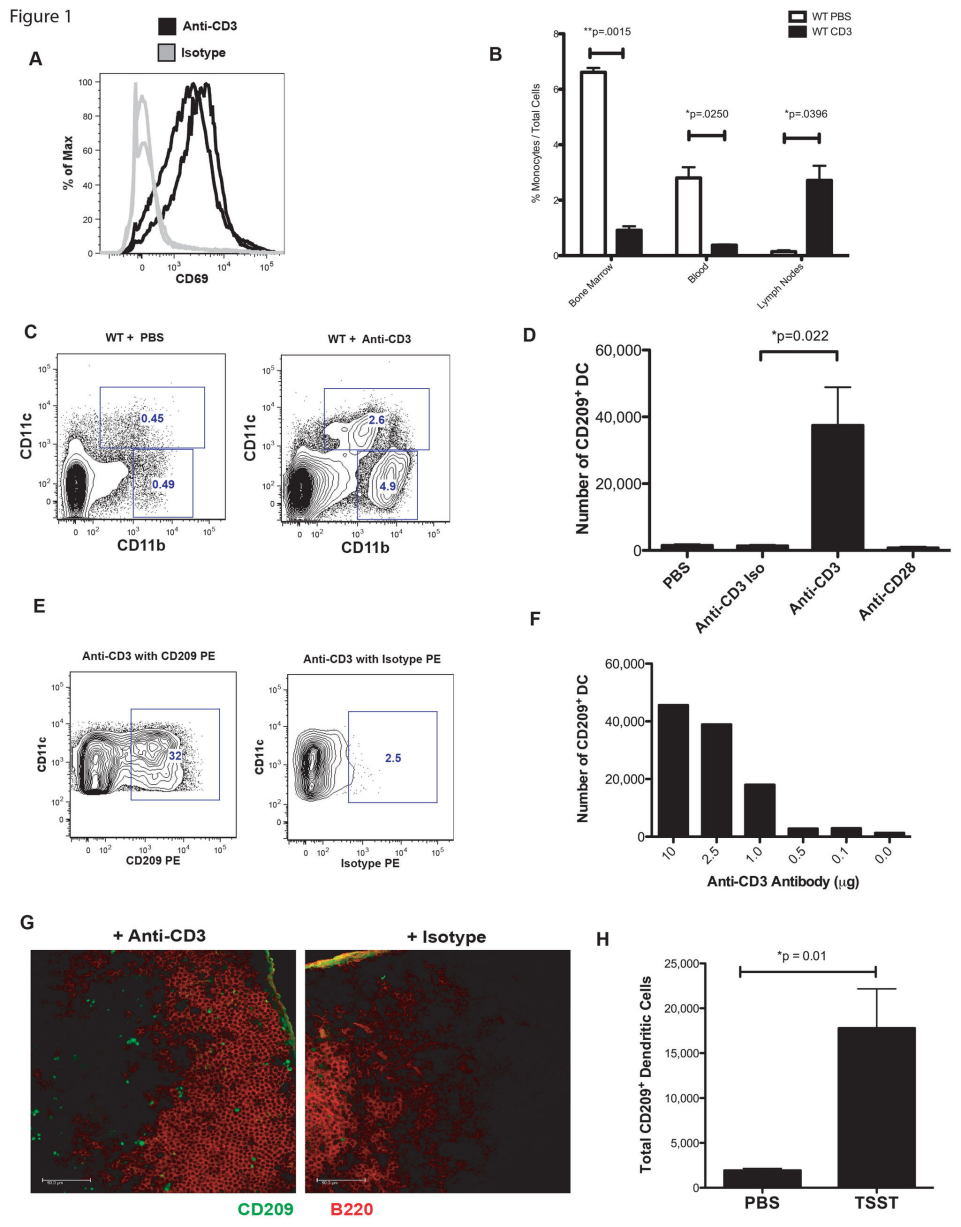
T cell activation mobilizes monocytes into LNs and promotes formation of CD209^+^ DC. A) Mice were injected in the footpad with 10µg anti-CD3 or an isotype control antibody. 18 hours later cells from the draining LNs were stained for the early T cell activation marker CD69. Cells shown were gated on DAPI^-^, Thy1.2^+^ cells. N=2 per group. Both duplicates are shown. B) Mice were injected in the footpad with 10µg anti-CD3 or PBS. 18 hours later, bone marrow, cardiac blood or draining LNs were stained for DAPI^-^, side scatter ^-/int^, CD11b^+^, CD11c^-^, Ly6c^+^ monocytes. Shown are mean cell percentage and SEM, N=2 mice per group. C) Mice were injected in the footpad with 10µg anti-CD3 or PBS. 18 hours later, draining LNs were evaluated for the frequency of CD11b^+^ CD11c^+^ DC or CD11b^+^ CD11c^-^ monocytes. Shown is one example representative of more than 5 individual experiments. D) Mice were injected in the footpad with 10µg anti-CD3, isotype control, anti-CD28 or PBS. The absolute number of CD209^+^, CD11b^+^, CD11c^+^, MHC II^+^, CD40^+^, Ly6c^-^, DAPI^-^ DC from 3 draining LNs is shown. Shown are mean cell number and SEM, N=4, representative of more than 5 individual experiments. E) Shown is one example of CD209 staining compared to isotype control staining of LN CD11c^+^, CD11b^+^ DC 18 hours after anti-CD3 antibody injection. The results shown are representative of 2 independent experiments. F) Mice were injected in the footpad with the indicated amounts of anti-CD3 antibody. 18 hours later the absolute number of LN CD209^+^ DC was determined with flow cytometry. G) Mice were injected in the footpad with 10µg anti-CD3 or an isotype control. 18 hours later draining LNs were removed, fixed and frozen. The sections were then stained for the presence of CD209 and B220. The scale bar is 50µM. H) Mice were injected IV with 25µg TSST-1 or PBS. 16 hours later, both inguinal and brachial LNs were harvested and the absolute number of CD209^+^, CD11b^+^, CD11c^+^, MHC II^+^, CD40^+^, Ly6c^-^, DAPI^-^ DC are shown as mean and SEM, N=4 mice per group.

To assess the effect of T cell activation on monocytes *in vivo*, we injected mice with a functional grade anti-CD3 (clone 2C-11) antibody to induce T cell activation. As expected and reminiscent of the effect of anti-CD3 (Clone OKT3) in humans [14], this antibody induced a marked increase in the expression of the early T cell activation marker CD69 on T cells (Figure **1A**). As shown in Figure **1B**, 18 hours following anti-CD3 injection, the frequency of monocytes (DAPI^-^, side scatter^lo/int^, CD11b^+^, CD11c^-^, Ly6c^+^) in the bone marrow and blood of anti-CD3 injected mice was significantly reduced compared to that of control mice injected with PBS. In contrast, there was a 10-fold increase in the frequency of monocytes in LNs (Figure **1C**). There was also a large increase in the number of CD11c^+^ DC in these LNs, but the origin of these DC was unclear.

As has been shown with a proprietary anti-CD209 clone [15], a commercially available anti-CD209 antibody (clone 5H10 from eBioscience) also effectively labels DC and not monocytes [11]. To determine the frequency of CD209^+^ Mo-DC formation following T cell activation, mice were injected intravenously with 10µg of dialyzed PE-conjugated anti-CD209 antibody and immediately thereafter 10µg of anti-CD3 activating antibody was administered by footpad injection. Twenty-four hours later, the draining LNs were removed and analyzed by flow cytometry to determine the frequency of CD209^+^ Mo-dC.T cell activation resulted in a highly significant increase in the number of CD209^+^ DC in the LNs (Figure **1D**). The increase in CD209^+^ Mo-DC required T cell activation, as an isotype control antibody for anti-CD3, or an anti-CD28 antibody that binds T cells but does not activate them had no effect on CD209^+^ Mo-DC number. These DC did not acquire the anti-CD209 antibody non-specifically, as they did not bind a PE-conjugated isotype control antibody (Figure **1E**). Further, the absolute number of DC in the LNs was highly correlated with the amount of anti-CD3 antibody injected (Figure **1F**).

The presence of CD209^+^ Mo-DC in the LNs was confirmed by immunofluorescence staining of frozen tissue from anti-CD3 treated mice, using a different anti-CD209 mAb (Figure **1G**). Interestingly, the CD209 staining appeared to be localized primarily within vesicles of Mo-DC, whereas the previously published CD209 staining of Mo-DC formed after LPS injection appeared to be primarily surface bound [15]. The CD209^+^ Mo-DC were present inside the B220^-^ T cell zone of LNs, consistent with the hypothesis that they may be involved in regulating T cell responses.

To ensure that this phenomenon was due to T cell activation and not to an off-target effect of the anti-CD3 antibody, we studied the effect of a bacterial super-antigen, toxic shock syndrome toxin-1 (TSST-1), on the formation of CD209^+^ Mo-DC. TSST-1 activates T cells by cross-linking MHC determinants with the T cell receptor (TCR) leading to the rapid secretion of T cell cytokines including IL-2, TNFα and IFNγ [16] and the expression of CD40L [17]. Interestingly, whereas injection of the super antigens SEB and SEA can result in T cell anergy, injection of TSST-1 does not [18]. We injected mice intravenously with 25µg of TSST-1 and examined the LNs 18 hours later. There was a 9-fold increase in the number of CD209^+^ Mo-DC compared to PBS treated control mice (Figure **1H**). These data demonstrate that robust T cell activation *in vivo* with either anti-CD3 or TSST-1 results in the differentiation of monocytes into cells with a DC phenotype.

### T cell induced CD209^+^ Mo-DC stimulate T cell proliferation, but do not elicit T cell polarization

**Figure 2:**
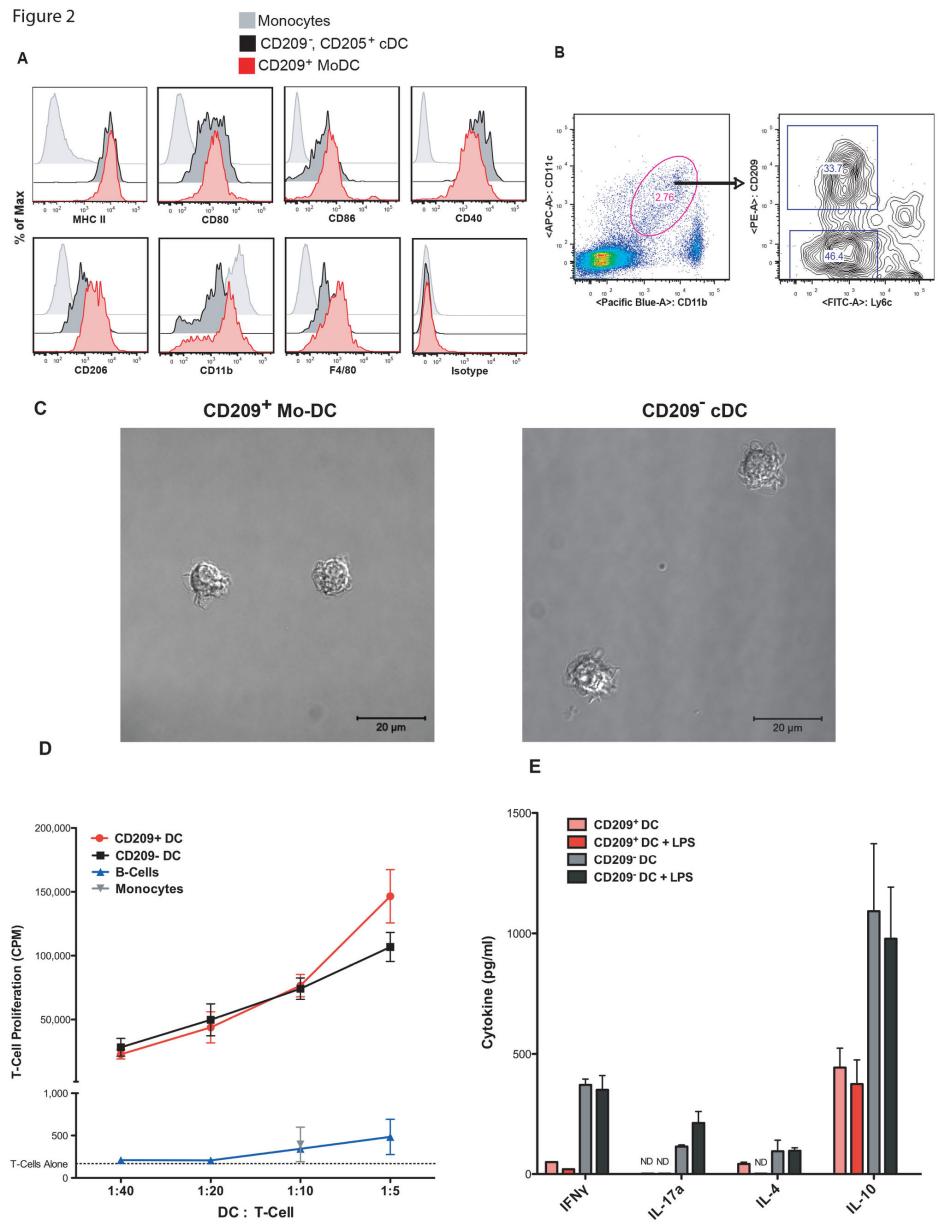
Activated T cell driven CD209^+^ Mo-DC stimulate T cell proliferation, but do not induce T cell polarization. A) Mice were injected in the footpad with 10µg anti-CD3. 18 hours later, LN monocytes gated as Ly6c^+^, CD11c^-^, side scatter^lo/inT cells^, Mo-DC gated as Ly6c^-^, CD11c^+^, CD209^+^, CD205^-^ cells and cDC gated as Ly6c^-^, CD11c^+^, CD209^-^, CD205^+^ cells were evaluated for expression of various surface proteins. Shown is a representative example from one mouse. B) The gating scheme for sorting CD209^+^ Mo-DC and CD209^-^ cDC is shown. C) DC sorted as in B) were plated on cover slip bottom chamber slides in medium and immediately imaged. The scale bar is 20µM. D) Cells sorted as in B) along with monocytes and B-cells were pulsed with 2.5µg/ml MHC class II restricted OVA peptide ISQ for 90 minutes at 37°C. They were then washed twice and plated at various ratios with OT-II CD4^+^ T cells from RAG KO mice. After 72 hours the cells were pulsed with H^3^-thymidine and harvested 18 hours later. Results of triplicate cultures are shown as mean and SEM, and are representative of 3 independent experiments. E) Cultures were set up as in D) except in some conditions 1µg/ml LPS was added during pulsing with peptide. After 72 hours, the cell-free supernatant was harvested and cytokines were measured by ELISA. Results are shown as mean and SEM, performed in triplicate from the 1 DC : 20 T cells condition, and are representative of 3 independent experiments. ND = not detected.

To further characterize the T cell induced CD209^+^ Mo-DC, we compared their cell surface phenotype with that of CD209^-^, CD205^+^ cDC and Ly6C^+^, CD11c^-^ monocytes from the same mice (Figure **2A**). CD209^+^ Mo-DC and CD209^-^ cDC expressed similar levels of MHC II and the costimulatory molecules CD80, CD86 and CD40. These levels were many-fold higher than found on monocytes. However, the CD209^+^ Mo-DC expressed higher levels of CD206, CD11b and F4/80 than cDC, suggesting that these Mo-DC may differ functionally from cDC.

To compare the immunological properties of CD209^+^ Mo-DC and CD209^-^ cDC, we sorted these cells from the LNs 18 hours after T cell activation based on their differential expression of CD209 (Figure **2B**). The cells were imaged with standard DIC and time-lapse microscopy (Figure **2C**). Both the CD209^+^ and CD209^-^ DC displayed wispy dendrites, which could be seen in constant motion via time-lapse microscopy (Movie **S1** and Movie **S2**). The DC subsets were subsequently pulsed with the MHC class II restricted OVA peptide ISQ in the absence of additional exogenous stimuli and cultured with CD4^+^ OVA specific OT-II T cells for 72 hours. CD209^+^ and CD209^-^ DC were equally effective at inducing T cell proliferation and were more than 100 times more potent than the same number of monocytes or B-cells (Figure **2D**). Whereas large amounts of IFNγ, IL-4, IL-17A and IL-10 were present in the T cell/cDC (CD209^-^) cultures, indicative of Th1, Th2, Th17 and Treg cell polarization, respectively, much less cytokine was detected in the T cell/Mo-DC (CD209^+^) cultures (Figure **2E**). Addition of LPS, a strong stimulus of DC maturation, to the cultures did not result in an increase in cytokine production. Thus, CD209^+^ T cell induced Mo-DC are efficient at presenting antigen and activating CD4^+^ T cells, but deficient in their ability to polarize these cells into the canonical Th1, Th2 and Th17 subtypes.

### CD40L, but not GM-CSF, contributes to CD209^+^ Mo-DC formation

**Figure 3:**
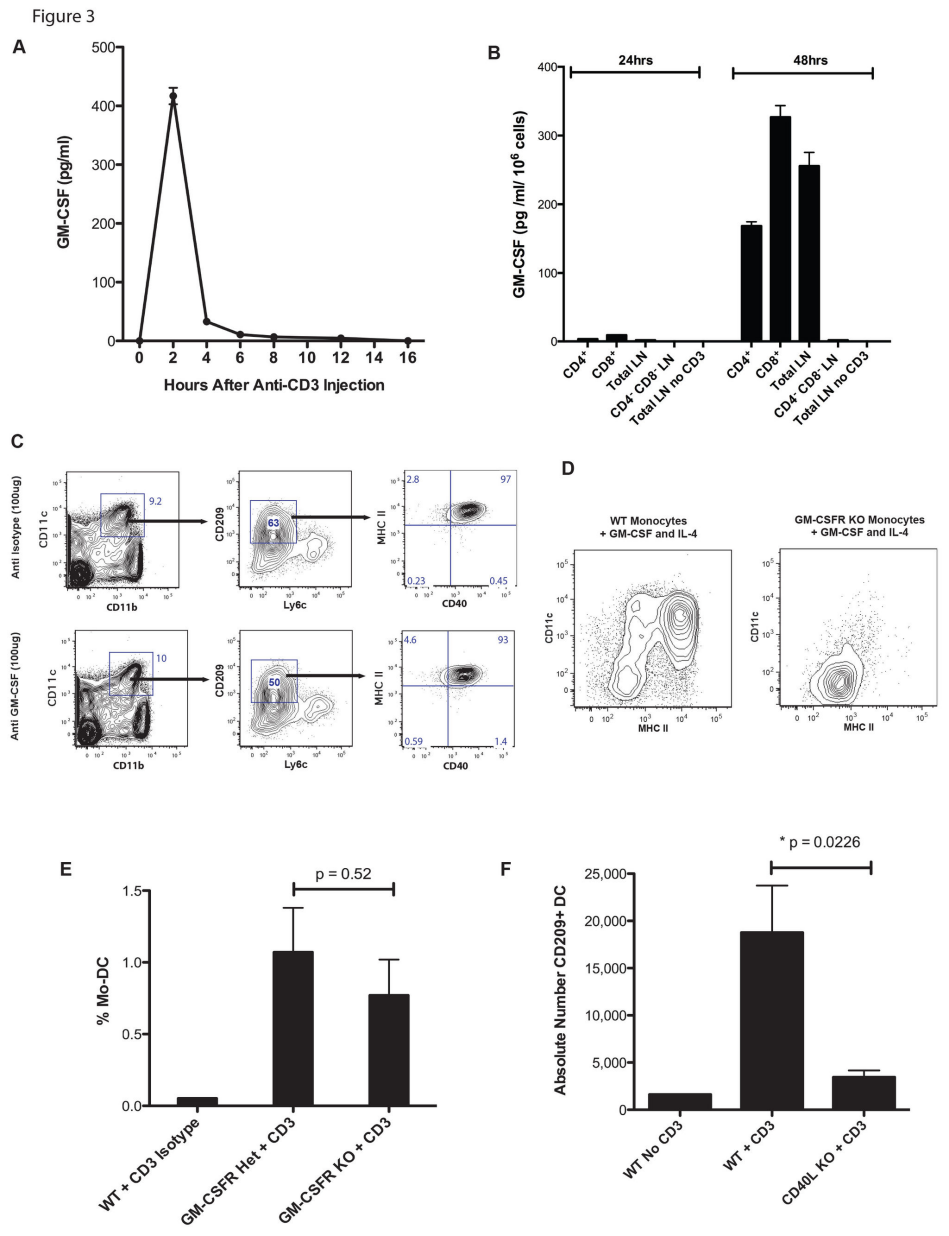
CD40L but not GM-CSF is needed for CD209^+^ Mo-DC formation. A) Serum from cardiac blood was assayed for GM-CSF at various times after footpad injection of 10µg anti-CD3 antibody. N=2 mice per time point. Results are shown as mean and SEM. B) CD4^+^ and CD8^+^ T cells were positively selected from the LNs of wild-type mice using biotinylated antibodies and anti-biotin microbeads. CD4^-^ CD8^-^ negative LN cells were also collected via negative selection. 1 x 10^6^ cells were cultured in triplicate in plates that were previously coated with 1µg/ml anti-CD3 antibody. Cell free supernatant was harvested after 24 and 48 hours for ELISA. C) Mice were injected IP with 100µg anti-GM-CSF blocking antibody or isotype control antibody. 4 hours later, the mice were injected in the footpad with 10µg/ml anti-CD3. 18 hours later the frequency of CD209^+^, CD11b^+^, CD11c^+^, MHC II^+^, CD40^+^, Ly6c^-^, DAPI^-^ Mo-DC was determined. Cells shown were first gated on Thy1.2^-^, CD19^-^, DX5^-^ cells. N=2 mice per group. The data shown are representative of 2 independent experiments. D) Bone marrow monocytes from wild-type or GM-CSFR KO mice were cultured with 50µg/ml GM-CSF and 20µg/ml IL-4 for 5 days. The cells were then analyzed by flow cytometry. Cells shown were gated on DAPI^-^, CD1lb^+^ cells. The data shown are representative of 2 independent experiments, 1 mouse per group. E) GM-CSFR heterozygous and GM-CSFR KO mice were injected in the footpad with 10µg anti-CD3. 18 hours later the frequency of CD209^+^, CD11b^+^, CD11c^+^, Mo-DC in the draining LNs was determined and the results are shown as the percentage of live LN cells. N=2 mice per group. F) Wild-type and CD40L KO mice were injected in the footpad with 10µg anti-CD3. 18 hours later the absolute number of CD209^+^, CD11b^+^, CD11c^+^ Mo-DC in the draining LNs was determined with flow cytometry. N=4 mice per group.

We next investigated the mechanism responsible for CD209^+^ Mo-DC formation after T cell activation. We hypothesized that GM-CSF produced by activated T cells may contribute to Mo-DC formation, as this cytokine is well known to drive Mo-DC formation and T cells can produce it after activation [19]. A time course experiment showed that GM-CSF was present in the serum of mice 2 hours following administration of the anti-CD3 antibody (Figure **3A**), and CD4^+^ and CD8^+^ T cells isolated from the LNs of naïve mice produced GM-CSF when activated *in vitro* with the same anti-CD3 antibody used *in vivo* (Figure **3B**). Moreover, LNs depleted of CD4^+^ and CD8^+^ T cells did not produce any detectable GM-CSF, indicating that these cells are the sole source of GM-CSF in LNs following anti-CD3 administration. However, when mice were injected with 100µg of a GM-CSF blocking antibody or an isotype control antibody, together with anti-CD3, there was no significant difference in the frequency of CD209^+^ Mo-DC at 24 hours in the draining LNs (Figure **3C**). It is possible that GM-CSF is produced in an immunological synapse or in some other manner rendering it inaccessible to our anti-GM-CSF antibody. To address this possibility, we utilized GM-CSFR KO mice that are unable to respond to GM-CSF (Figure **3D**). Following injection of anti-CD3 antibody into groups of GM-CSFR heterozygous and KO mice, the extent of Mo-DC formation was similar in both groups (Figure **3E**), demonstrating that signaling though the GM-CSFR is not necessary for the formation of Mo-DC following T cell activation. Thus, although GM-CSF can induce the differentiation of monocytes into DC, it is not required for the formation of CD209^+^ Mo-DC after T cell activation.

We next investigated the role of CD40L in this process. Unlike most T cell cytokines CD40L is expressed within minutes of T cell activation, due to the presence of preformed CD40L in secretory lysosomes, and is then maintained via protein synthesis [20]. As shown in Figure **3F**, mice lacking CD40L had far fewer CD209^+^ DC in their LN compared to wild-type controls following injection of anti-CD3 antibody. This result demonstrates a critical role for CD40L in the development of CD209^+^ Mo-DC.

### Upregulation of PDL-2 on CD209^+^ Mo-DC hinders their ability to polarize naïve T cells

**Figure 4:**
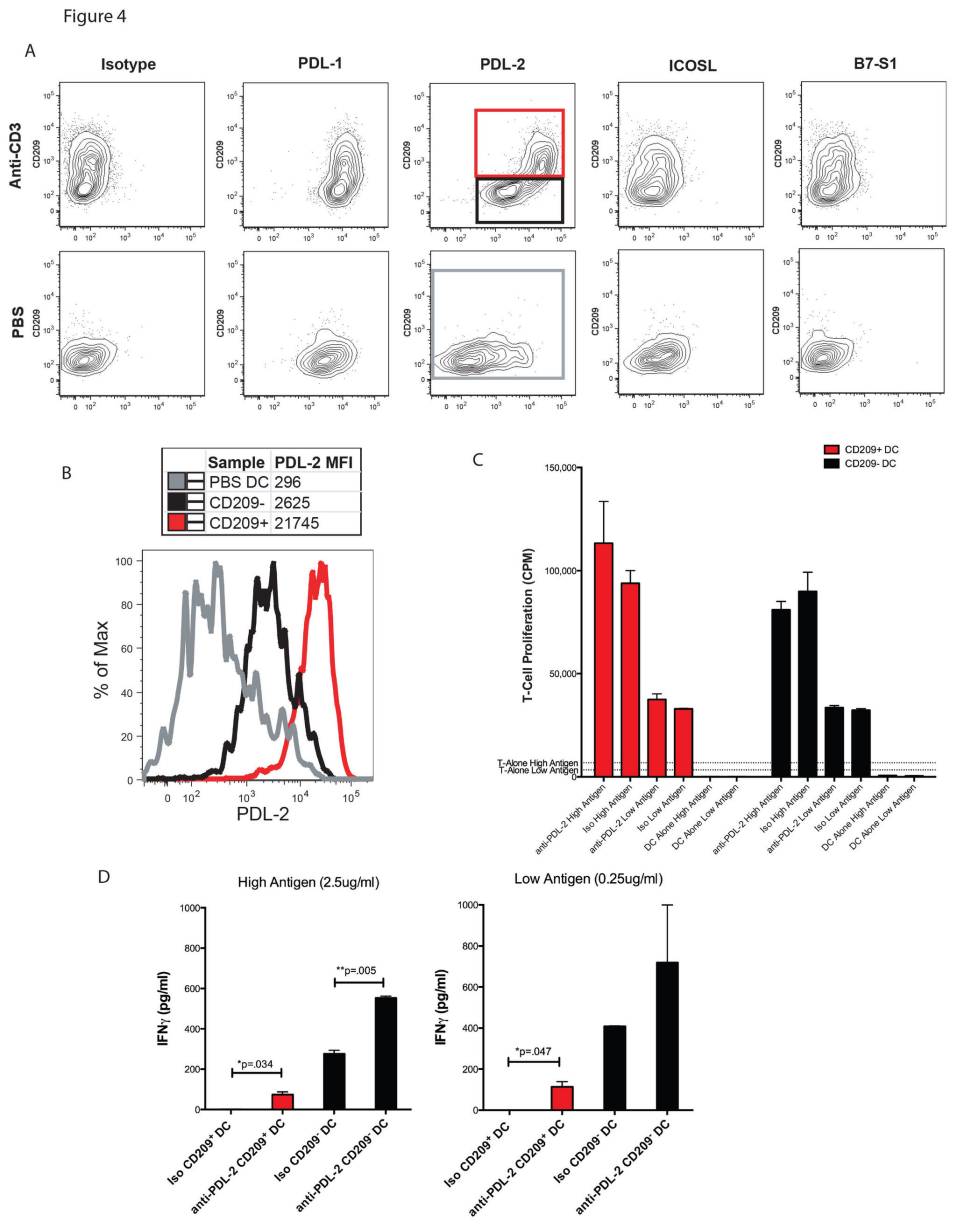
Upregulated PDL-2 expression inhibits CD209^+^ Mo-DC from polarizing naïve T cells. A) Mice were injected in the footpad with 10µg anti-CD3 or PBS. 18 hours later, the draining LNs were evaluated for the expression of alternative costimulatory molecules on CD11c^+^, CD11b^+^, Ly6c^-^ DC subsets. Results shown are representative of 2 independent experiments. Red (Mo-DC [CD209^+^] from anti-CD3 treated mice), black (cDC [CD209^-^] from anti-CD3 treated mice) and gray (total DC from PBS treated control mice). B) The DC subsets shown in boxes in A) were evaluated for PDL-2 expression (MFI = median fluorescence intensity). C) CD209^+^ and CD209^-^ DC were sorted and cultured at a 1:10 ratio with naïve CD4^+^ T cells from OT-II RAG KO mice in the presence of high (2.50µg/ml) or low (0.25µg/ml) ISQ peptide and 10µg/ml anti-PDL-2 antibody or an isotype control antibody. After 72 hours, the cells were pulsed with H^3^-thymidine and harvested 20 hours later. The results of triplicate cultures are shown as mean and SEM, done in triplicate. D) Aliquots of the supernatants from the cultures in C) were removed after 72 hours of culture and tested for IFNγ by ELISA. The results of triplicate cultures are shown as mean and SEM. Iso refers to isotype control antibody.

Many alternative costimulatory molecules in addition to CD80 and CD86 have been shown to modulate T cell stimulation and polarization [21]. Because CD209^+^ Mo-DC resemble cDC in their morphology and expression of MHC II and classical costimulatory molecules, we hypothesized that they may express different alternative costimulatory molecules rendering them unable to induce T cell polarization. CD209^+^ and CD209^-^ DC from anti-CD3 treated or control mice were evaluated for expression of PDL-1, PDL-2, ICOSL and B7-S1 (Figure **4A**). PDL-1 was slightly upregulated on both DC subsets after anti-CD3 stimulation, while ICOSL and B7-S1 remained unchanged. On the other hand, PDL-2 was upregulated by 9-fold on CD209^-^ DC and by more than 70-fold on CD209^+^ Mo-DC compared to DC from control mice (Figure **4B**).

To investigate the role of PDL-2 in the functions of anti-CD3 induced Mo-DC, we sorted DC into CD209^+^ and CD209^-^ subsets and cultured the cells with low (0.25µg/ml) or high (2.5µg/ml) amounts of OVA peptide, along with OT-II T cells and a functional grade blocking or isotype control antibody to PDL-2. As shown in Figure **4C**, anti-PDL-2 had no effect on the stimulatory capacity of either DC subset, regardless of antigen concentration. To examine the possibility that blockade of PDL-2 might enable the CD209^+^ DC to polarize T cells, we evaluated the culture supernatant from the previous experiment for the presence of IFNγ (Figure **4D**). At both high and low antigen concentrations, blockade of PDL-2 reestablished the ability of CD209^+^ DC to induce IFNγ production, albeit at levels lower than that produced by T cells cultured with CD209^-^ DC. Anti-PDL-2 also enhanced the induction of IFNγ by CD209^-^ DC. These data demonstrate that PDL-2 is highly expressed on CD209^+^ Mo-DC following T cell activation and helps prevent these cells from polarizing, but not activating, CD4^+^ T cells.

## Discussion

This study demonstrates that T cell activation alone, in the absence of PAMPs, is sufficient to elicit monocyte differentiation into CD209^+^ Mo-DC, *in vivo*. Although T cell induced DCs were found in LNs, their differentiation from monocytes did not necessarily occur there. Under inflammatory conditions, monocytes from bone marrow and blood infiltrate tissues where they can encounter activated T cells. In this regard, our previous studies of inflamed skin in patients with psoriasis and atopic dermatitis revealed frequent multipoint interactions between activated T cells and monocytes, as well as clusters of T cells, monocytes and DCs, suggesting that DC differentiation from monocytes occurs at such sites. Whether in the current studies DCs differentiated in non-lymphoid tissues and then migrated to draining LNs, or differentiated from monocytes in LNs, is unknown, since our data are consistent with either possibility. We found that CD40L is required for optimal generation of CD209^+^ Mo-DC. While soluble CD40L can be released *in vivo*, it seems more likely that chemokines such as CCL2 recruited monocytes to tissues where interaction with CD40L^+^ T cells directed their differentiation into CD209^+^ Mo-DC.

The formation of CD209^+^ Mo-DC correlated with the intensity of T cell activation. Interestingly, Mo-DC formation was not observed with anti-CD3 antibody doses less than 1µg, indicating that a minimum threshold of T cell activation was required. Such a threshold is likely exceeded during infection and sterile inflammatory disorders such as graft versus host disease (GVHD) and transplant rejection, where both T cells and DC are believed to play a critical pathogenic role [22-24]. It is plausible that monocytes serve as a pool of reserve DC that form during acute or sustained inflammation. Whereas certain microbial products can drive such DC formation, so can activated T cells whose specificity and memory for past infections make them ideally suited for this purpose. On the other hand, if the T cell stimuli are not eliminated, as might occur in the setting of GVHD or sustained reactivity against self, Mo-DC may contribute to tissue-damaging inflammation.

Although in our study, CD209^+^ DC were potent inducers of T cell proliferation, they were severely impaired in their ability to polarize naïve T cells, as indicated by the scarcity of IFNγ, IL-17A or IL-4 in the supernatants of CD209^+^ Mo-DC and T cell co-cultures. The inability of these cells to polarize T cells is partly explained by their increased expression of the alternative costimulatory molecule PDL-2, since blockade of this molecule restores some polarizing capacity to the CD209^+^ Mo-DC. There are conflicting reports on the function of PDL-2, which is also known as B7-DC and CD273. While some reports claim that this molecule is stimulatory [25], the majority indicate that PDL-2 has an immunosuppressive effect by signaling though PD-1 and potentially other unidentified receptors on T cells [26-28]. Importantly, at least one study suggests that the immunosuppressive effect of PDL-2 on T cells depends on the amount of antigen and thus the strength of the activation signal provided to the T cells [29,30].

Our data indicate that PDL-2 molecules on T cell induced Mo-DC act as a brake on T cell polarization, but not T cell proliferation. How PDL-2 regulates T cell polarization is not known, but based on studies with PDL-1, it appears that the intensity of PD1 signaling is critical in the regulation of multiple cytokines [31]. It is possible that PDL-2 signaling behaves similarly, as it is a known ligand for PD1. However, other explanations are possible, since PDL-1 causes a conformational change in PD-1 upon binding, but PDL-2 does not [32] and both ligands compete for the same receptor, yet have different affinities. Therefore, one effect of high PDL-2 expression might be a decrease in PDL-1 mediated signaling [33]. Interestingly, there are data demonstrating that PDL-2 has biological activity even when PD-1 binding is abolished, suggesting there are yet unidentified pathways by which PDL-2 may be modulating T cell polarization [34].

The limited T cell polarizing activity of CD209^+^ Mo-DC presumably mitigates the risk that these cells promote or enhance inflammation. On the other hand, in addition to reduced levels of proinflammatory cytokines in the supernatants of the Mo-DC and T cell cultures, the amount of IL-10 produced in these cultures was also lower than that produced in cDC and T cell co-cultures. These results suggest that T cells stimulated by CD209^+^ Mo-DC probably rely on other cues for their polarization. In the absence of such cues, these newly activated T cells may take on an IL-10 secreting immunosuppressive phenotype and serve as a brake on inflammation. This notion of specific DC subsets serving to inhibit massive inflammatory responses has been reported with specialized DC capable of producing the tryptophan catabolizing enzyme indoleamine 2,3-dioxygenase (IDO) after being exposed to large amounts of CpG DNA *in vivo* [35]. Based on these results, we speculate that CD209^+^ Mo-DC elicited by activated T cells may play a proinflammatory or anti-inflammatory role *in vivo*, depending on the nature of the signals they receive.

## Supporting Information

Movie S1 **Time-lapse movie of CD209^+^ DC.** Freshly sorted DC were plated onto coverslip bottom chamber slides in media and imaged. 20 minutes of microscopy was acquired at a rate of 1 frame every 10 seconds. The data is presented as a movie shown at 100X actual speed.
(MOV)

Movie S2 **Time-lapse movie of CD209^-^ DC.** Freshly sorted DC were plated onto coverslip bottom chamber slides in media and imaged. 20 minutes of microscopy was acquired at a rate of 1 frame every 10 seconds. The data is presented as a movie shown at 100X actual speed.
(MOV)
